# Trifolirhizin improves the hyperproliferation and excessive inflammatory response in human HaCaT keratinocytes and ameliorates skin lesions in psoriasis-like mouse models

**DOI:** 10.1590/1414-431X2025e14766

**Published:** 2025-08-22

**Authors:** Linyu Zhu, Menger Guo, Ling Wang, Shaomin Chen, Zhiyu Ye, Yuansheng Wu

**Affiliations:** 1 Department of Dermatovenereology, The Second Clinical College of Guangzhou University of Chinese Medicine, Guangzhou, China; 2 Department of Dermatovenereology, The Seventh Affiliated Hospital of Sun Yat-sen University, Shenzhen, China; 3 Department of Traditional Chinese Medicine, The Seventh Affiliated Hospital of Sun Yat-sen University, Shenzhen, China; 4 Department of Dermatovenereology, Guangdong Provincial Hospital of Chinese Medicine, Guangzhou, China

**Keywords:** Psoriasis, Keratinocytes, Trifolirhizin, Autophagy, Inflammatory factors

## Abstract

Keratinocyte hyperproliferation and excessive inflammatory responses are associated with psoriasis pathogenesis. Trifolirhizin has anti-inflammatory and anti-proliferation effects. The purpose of the study was to investigate the role of trifolirhizin in psoriasis-like skin lesions and its molecular mechanism. Imiquimod-induced psoriasis-like mouse models were treated with trifolirhizin. Skin lesions and inflammatory factors were assessed. *In vitro*, human HaCaT keratinocytes were stimulated by a mixture of interleukin (IL)-1α, IL-17, IL-22, tumor necrosis factor (TNF)-α, and oncostatin M (M5) to establish a psoriatic keratinocyte model. Cell viability and cycle were assessed via CCK-8 assay and flow cytometry. Inflammatory factors, autophagy levels, and AMPK-mTOR pathway activation were detected by western blot. Trifolirhizin dose-dependently inhibited epidermal layer erythema, scaling, and thickening and reduced epidermal thickness and IL-12 level in an imiquimod-induced psoriasis-like mouse model. Trifolirhizin also inhibited cell viability, PCNA expression, and excessive synthesis and secretion of IL-8 and IL-12 in HaCaT keratinocytes induced by M5. Furthermore, the inhibition of autophagy and AMPK-mTOR pathway could be reversed by trifolirhizin in M5-induced HaCaT keratinocytes and skin lesions from imiquimod-mediated psoriasis-like mouse model. The improvement effects of trifolirhizin could be inhibited by the autophagy inhibitor chloroquine. Trifolirhizin up-regulated autophagy through the AMPK-mTOR pathway, improved the hyperproliferation and excessive inflammatory responses of keratinocytes, thus alleviating psoriatic skin lesions. Trifolirhizin may have therapeutic potential in improving the progression of psoriasis.

## Introduction

Psoriasis is a common chronic inflammatory and hyperproliferative skin disorder characterized by patches of thick, red skin covered with silvery scales, usually accompanied by itching and pain (1). The disorder affects approximately 1-3% of the population worldwide (2). Psoriasis is associated with multiple complications, ranging from respiratory to cardiovascular and gastrointestinal diseases (3,4). Although studies on the pathogenesis of psoriasis have been extensively developed, there is no known cure. The exact etiology and pathogenesis of psoriasis are still unclear. Therefore, it is urgent to explore new treatment strategies or methods for psoriasis.

The main pathological manifestations of psoriasis are the hyperproliferation of epidermal keratinocytes, the infiltration of inflammatory cells, and redirection and the dilation of vessels in the superficial dermis (1). Inhibiting the hyperproliferation of keratinocytes can improve psoriatic lesions (5,6). In addition, the excessive activation of the immune system plays important roles in the pathogenesis of psoriasis. For instance, in psoriasis, Th1 cells can secrete tumor necrosis factor (TNF)-α, while Th22 cells secrete interleukin (IL)-22, and Th17 cells secrete IL-17, IL-22, and TNF-α (7). These cytokines can then promote proliferation and the expression of cytokines, including IL-8 and IL-12, in keratinocytes (7-9). TNF-α can activate myeloid dendritic cells and enhance the secretion of IL-12 and IL-23. IL-12 and IL-23 are central to the survival, proliferation, and activation of Th17 cells (7,10), resulting in an inflammatory cascade that leads to psoriatic disease manifestations. Furthermore, IL-8 has a chemotaxis effect on neutrophils and T lymphocytes, and it can promote the production of new vessels and proliferation of keratinocytes (11). Therefore, inhibition of hyperproliferation and excessive secretion of IL-8 and IL-12 of keratinocytes is a potential therapeutic strategy for psoriasis.

Although biologics that inhibit TNF-α, p40IL-12/23, IL-17, and p19IL-23, as well as an oral phosphodiesterase 4 inhibitor, represent a therapeutic advance in moderate to severe plaque psoriasis, these biologics are associated with numerous adverse events, including nasopharyngitis, upper respiratory tract infections, and injection site reactions (7). Traditional Chinese medicine (TCM) has a long history of use in China and other Asian countries for treating and preventing human diseases and is part of mainstream medicine in these countries. The use of herbal medicines to prevent the development and recurrence of psoriasis is widely accepted (12). *Sophora flavescens*, a Chinese herbal medicine, has a long clinical history for the treatment of various human illnesses such as non-small cell lung cancer, hepatitis B infections, and liver fibrosis (13). Topical multi-herbal formulations containing *Sophora flavescens* have been reported to improve overall clinical symptoms of psoriasis (14). However, there are many compounds in *Sophora flavescens* and the mechanism by which *Sophora flavescens* alleviates psoriasis is unknown. Trifolirhizin is a pterocarpan flavonoid isolated from *Sophora flavescens*, with a variety of pharmacological effects, such as anti-inflammatory (15) and anti-proliferative (16). For example, trifolirhizin can inhibit the proliferation of gastric cancer cells and improve the progression of gastric cancer (17). The role of trifolirhizin in psoriatic skin lesions, hyperproliferation, and excessive inflammatory factors of keratinocytes in psoriasis needs to be confirmed.

Animal and cellular models that can simulate clinical psoriasis lesions are very important to explore the efficacy of trifolirhizin in psoriasis. It has been reported that imiquimod (IMQ), an agonist of Toll-like receptor 7/8 ligand, induces dermatitis in mice closely resembling human psoriasis (18). Thus, IMQ is widely used to stimulate BALB/c mice to induce psoriasis-like lesions (19). Hyperproliferation is related to excessive secretion of pro-inflammatory factors such as IL-17A in the epidermis (20). In addition, IL-17A, interferon (IFN)-γ, and other factors can also promote keratinocyte proliferation and secretion of IL-8 (8) and IL-12 (21). Consequently, the cocktail of IL-17A, IL-22, oncostatin M, IL-1α, and TNF-α (M5) induces keratinocytes to manifest some psoriatic features *in vitro* establishing a psoriatic keratinocyte *in vitro* model (19,22,23). In this study, we investigated the effects of trifolirhizin on psoriasis-like skin lesions, hyperproliferation, and excessive inflammatory responses of keratinocytes, and explored the underlying mechanism.

## Material and Methods

### Animal grouping

BALB/c mice (male, about 7 weeks old) were purchased from Hubei Experimental Animal Research Center (SCXK (Hubei) 2020-0018). Follow-up experiments were conducted after one week of adaptive feeding. BALB/c mice were divided into five groups with six mice each, and four groups were used to construct the psoriasis model.

After removing the skin hair on the back, 50 mg of cream containing 5% IMQ (An Lehui, Ganweixiao Zi (2019) No. D049, China) was applied daily for 5 days (24). The remaining group of mice was a normal control group, and the same amount of vehicle cream and an intraperitoneal injection of phosphate-buffered saline (PBS) was applied daily for 5 days after removing the skin hair on the back. The psoriasis-like mice were divided into four groups and treated with an intraperitoneal injection of PBS or different doses of trifolirhizin (Aladdin, 6807-83-6, China): 10, 20, and 40 mg/kg, according to the literature (25). While applying IMQ, an intraperitoneal injection of PBS or trifolirhizin were given at the same time. All experimental protocols and procedures for this study were approved by the Institutional Animal Care and Use Committee of the Seventh Affiliated Hospital of Sun Yat-sen University and comply with the National Institutes of Health guidelines for the care and use of experimental animals.

### Scoring of skin inflammation severity

The back skin of each mouse was photographed at 1, 2, 3, 4, and 5 days after cream application and absorption. The severity of skin inflammation of mice with different treatments was scored using the clinical Psoriasis Area and Severity Index (PASI). Skin inflammation was evaluated based on erythema, scaling, and thickening, which were scored independently on a scale between 0 and 4 as follows: 0) none; 1) slight; 2) moderate; 3) marked; and 4) very marked. The cumulative score of erythema, scaling, and thickening was used to determine inflammation severity (scales 0-12) (24). The individuals scoring the skin inflammation severity were blinded to the treatment groups.

### ELISA

The epidermis was separated from the dermis with thermolysin (26). IL-12 was measured in the epidermal layer of the skin tissue of mice treated with different methods with a specific ELISA kit (E-EL-M3062, Elabscience, China). IL-8 and IL-12 were measured in the supernatant of HaCaT cells with different treatments with ELISA kits (E-EL-H6008 and E-EL-H0150c, Elabscience). The absorbance curves obtained for the samples were compared with standard curves to calculate the concentration of the samples.

### Cell culture and treatment

Human HaCaT immortalized keratinocytes purchased from ATCC cell bank in the United States were cultured in DMEM medium (Pricella, PM150210, China) containing 10% FBS (Excell, 12A243, China) in a 5% CO_2_ incubator at 37°C. Cells in the logarithmic growth stage were selected for various experiments. HaCaT cells were inoculated onto the culture plate with medium containing M5 and/or different concentrations of trifolirhizin (5, 10, 20, 40 μM; Aladdin, T139172) in the presence or absence of autophagy inhibitor chloroquine (CQ, 40 μM (27), Aladdin, C193834). The cells were treated for 48 h. M5 was composed of 10 ng/mL of IL-1α (MCE, HY-P72380, USA), IL-17 (Gibco, 200-17-1MG, USA), IL-22 (MCE, HY-P7039), TNF-α (Gibco, 300-01A-1MG), and oncostatin M (MCE, HY-P70465G).

### Detection of cell activity by CCK8

Commercial CCK-8 assay kit (MCE, HY-K0301) was used to detect the activity of HaCaT cells in each treatment group. HaCaT cells (3×10^3^/well) were inoculated onto 96-well cell culture plates and cultured overnight in a 5% CO_2_ incubator at 37°C. The cells were treated with the different treatments. After 48 h, 10 μL CCK8 was added to each well and cultured at 37°C for 2 h.

### Western blot

After the epidermal layer of skin tissue was cut or the cell culture medium was removed, the cells were lysed on ice for 30 min by the lysis buffer (Beyotime, P0013B, China) with PMSF (Nanjing Wohong, 329-98-6, China). The lysate was transferred to a 1.5-mL centrifuge tube. The sample was centrifuged at 13800 *g* at 4°C for 5 min and the supernatant was removed. After mixing the protein samples with 5× protein loading buffer (P0286, Beyotime), the samples were boiled in water for 10 min. Then, 40 μg protein sample and standard protein molecular weight marker (Thermo Scientific, 26612, USA) were added to the sample well, and the sample was separated at 80 V for 30 min and then 120 V for 1.5 h by electrophoresis. After membrane transfer and membrane blocking, the PVDF membrane was incubated with anti-PCNA antibody (1:1000; Proteintech, 18892-1-AP, China), anti-IL-8 antibody (1:1000; Affinity, DF6998, USA), anti-IL-12 antibody (1:1000; Proteintech, 60306-1-Ig), anti-LC3 antibody (1:1000; Affinity, AF5384), anti-p62 antibody (1:1000; Affinity, AF5384), anti-p-AMPK antibody (1:1000; Abcam, ab92701, UK), anti-AMPK antibody (1:2000; Abcam ab32074), anti-p-mTOR antibody (1:10, 000; Abcam, ab134903), anti-mTOR antibody (1:1000; Affinity, AF5384), or anti-GAPDH antibody (1:2500; Abcam, ab9485) overnight at 4°C. Then, the PVDF membrane was incubated with sheep anti-rabbit antibody labeled with HRP (1:3000, Beyotime, A0208) or sheep anti-mouse antibody labeled with HRP (1:3000, Beyotime, A0216) at 37°C for 1 h. Finally, the enhanced solution of ECL reagent and the stable peroxidase solution were mixed at a 1:1 ratio, the working solution was added to the PVDF membrane, and a gel imaging system (Biorad, ChemiDoc XRS+ System, USA) was used to scan the PVDF membrane.

### Cell cycle distribution analysis flow cytometry

HaCaT cells digested by trypsin were collected and centrifuged at 277 *g* for 5 min at room temperature. The supernatant was removed, and PBS was used to resuspend and rinse the cells twice. Pre-cooled 80% ethanol (700 μL) was slowly added to 100 μL cells suspended in PBS to make the final concentration of ethanol to 70%. The mixture was fixed at 4°C for more than 4 h and centrifuged at 433 *g* for 5 min at 4°C. The cells were washed with pre-cooled PBS twice and 200 μL cells suspended in PBS was prepared. RNase (10 μL, 1 mg/mL) was added, and the mixture was incubated at 37°C for 30 min. PI (10 μL, 400 μg/mL, Nanjing KGI, KGA108) was added to stain the cells at 4°C for 30 min avoiding light. Finally, the cells were analyzed by flow cytometry (BECKMAN, CytoFLEX, USA).

### qRT-PCR

One1 mL Trizol reagent was added to cells, and then the mixture was mixed well with a pipette. For fresh frozen tissue stored in the refrigerator at -80°C, 1 mL Trizol reagent was added to a part with a weight of about 100 mg and the tissue was ground into pulp with a homogenizer. The mixture was transferred to a 1.5-mL EP tube without RNase and lysed for 10 min. Then, chloroform was added. The mixture was shaken vigorously until emulsified (the solution is milky white), and then let stand at room temperature for 5 min. The mixture was centrifuged at 12000 *g* at 4°C for 15 min. The colorless supernatant was aspirated and precipitated by isopropyl alcohol. The precipitation was cleaned by ethanol and finally added to the RNA solution for precipitation and dissolution. The RNA reverse transcription to cDNA was performed with reverse transcription kit (Fermentas, K1622, Canada), and PCR amplification was performed with the SYBR Green qPCR kit (Thermo, K0222, USA). Real-time fluorescence quantitative PCR (ABI, 7500, USA) was used for on-computer detection, and the data were normalized with internal parameters. Primers were as follows: IL-8: Forward: GACATACTCCAAACCTTTCCACCCC; Reverse: CAAAAACTTCTCCACAACCCTCTGC. IL-12: Forward: GATGGCCCTGTGCCTTAGTA; Reverse: TCAAGGGAGGATTTTTGTGG. GAPDH: Forward: TCAAGAAGGTGGTGAAGCAGG; Reverse: TCAAAGGTGGAGGAGTGGGT.

### Data analysis

Prism 9.0 statistical software (GraphPad, USA) was used for data analysis, and all the data are reported as means±SD. The comparison among different groups was performed using the LSD method (minimum significance method) in one-way ANOVA. P<0.05 was considered statistically significant.

## Results

### Trifolirhizin can ameliorate skin lesions and the protein level of inflammatory factor IL-12 in IMQ-induced psoriasis-like mouse models

**Figure 1:**
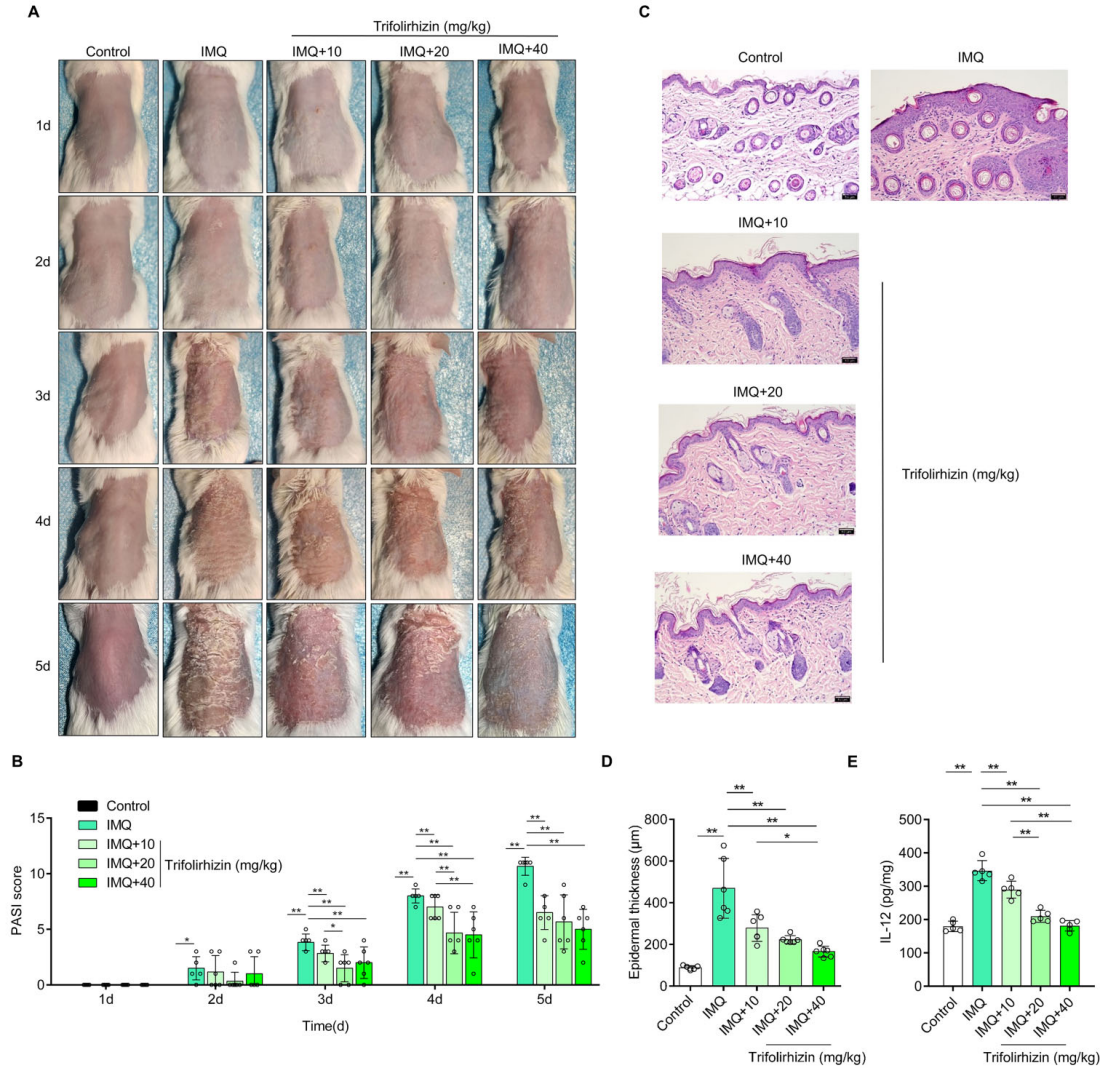
Effects of trifolirhizin on skin lesions and the contents of inflammatory factor interleukin (IL)-12 in epidermal tissue in imiquimod (IMQ)-induced psoriasis-like mouse models. The mice were treated with an intraperitoneal injection of phosphate-buffered saline or different doses of trifolirhizin (10, 20, or 40 mg/kg) after removal of the back hair, while applying vehicle cream or cream containing 5% IMQ, for 5 consecutive days. **A**, The back skin of mice was photographed at 1, 2, 3, 4, and 5 d after cream application. **B**, The scales, erythema, and thickness of the skin of mice were observed at 1, 2, 3, 4, and 5 d after cream application and absorption, and Psoriasis Area and Severity Index (PASI) score was calculated. **C**, HE staining. Scale bar=50 μm. **D**, Epidermal thickness. **E**, IL-12 level in the epidermal layer detected by ELISA. Data are reported as means±SD. *P<0.05, **P<0.01; ANOVA.

There was no erythema, scales, and thickening in the normal control mice smeared with vehicle cream, meaning that PASI score was 0. The back skin of mice smeared with IMQ showed obvious erythema, scales, and thickening, and the symptoms worsened with longer smeared time, i.e., PASI score increased to more than 10 at the end of the experiment (Figure 1A and B). After IMQ application, the mice showed obvious histopathological features of psoriasis, including inflammatory cell infiltration and the increase of epidermal thickness from 100 μm to more than 400 μm (Figure 1C and D). This demonstrated that the psoriasis-like mouse model was built successfully.

The low, medium, and high doses of trifolirhizin could improve the erythema, scales, and thickening caused by IMQ application, which was manifested as a decrease in PASI score. Compared to the low dose, the medium and high doses had stronger inhibitory effects on PASI score at 4 d. However, there was no significant difference between these three doses in terms of PASI score at the end of the experiment (Figure 1A and B). Furthermore, trifolirhizin reduced the thickness of the epidermal layer and inhibited the infiltration of inflammatory cells in the psoriasis mice. The improvement on epidermal layer thickness was stronger with the high dose of trifolirhizin than with the low dose. However, there was no significant difference in epidermal layer thickness between medium and high dose treatment groups (Figure 1C and D). In addition, IL-12 levels in the skin epidermis were increased in mice with IMQ, which was inhibited by three doses of trifolirhizin, and the inhibitory effects were enhanced with increasing concentrations. Compared to the low dose group, the inhibitory effects of the medium and high doses on IL-12 levels were stronger. The improvement of the medium dose was not significantly different from that of the high dose (Figure 1E). In conclusion, trifolirhizin improved skin lesions and IL-12 level in mice with psoriasis in a dose-dependent manner.

### Trifolirhizin inhibited hyperproliferation induced by M5 in human HaCaT keratinocytes

**Figure 2:**
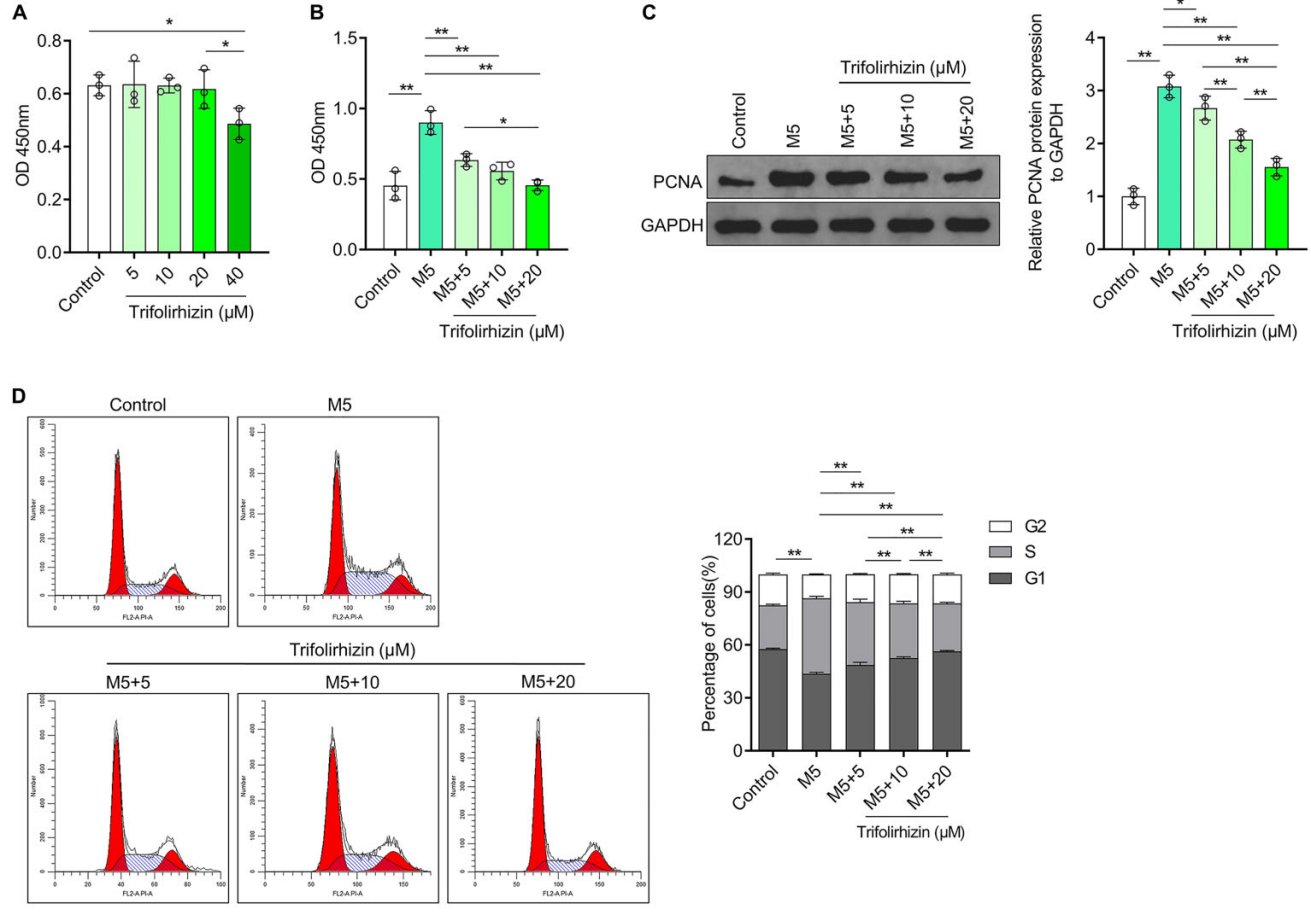
Effects of different concentrations of trifolirhizin (5-40 μM) on M5-induced human HaCaT keratinocytes hyperproliferation. **A**, The cell viability was detected by CCK8. **B** and **C**, HaCaT keratinocytes were treated with trifolirhizin at different concentrations (5-20 μM) and control medium or M5 medium for 48 h. **B**, Cell viability by CCK-8 assay. **C**, Western blot image and analysis of PCNA protein level using GAPDH as the internal reference. **D**, Cell cycle distribution (compared to G1 phase) analyzed by PI staining combined with flow cytometry. Data are reported as means±SD. *P<0.05, **P<0.01; ANOVA.

In order to determine the effect of trifolirhizin on psoriasis-like keratinocyte hyperproliferation, firstly, human HaCaT keratinocytes were treated with different concentrations of trifolirhizin to determine the effects of trifolirhizin on keratinocyte toxicity in a normal environment. CCK-8 test results showed that 5-20 μM trifolirhizin could not inhibit the cell viability, while 40 μM trifolirhizin could significantly inhibit the cell viability (Figure 2A). Therefore, in subsequent experiments, HaCaT keratinocytes were treated with 5-20 μM trifolirhizin together with M5. Cell viability and the expression of proliferation marker PCNA were increased, and the proportion of the cell cycle G1 phase was shortened in human HaCaT keratinocytes exposed to M5 accompanied by an increased proportion of S phase (Figure 2B-D). These results suggested that M5 could indeed induce keratinocyte hyperproliferation and could be used to simulate psoriasis-like keratinocytes. M5-mediated increase in cell viability of HaCaT keratinocytes was inhibited by three doses of trifolirhizin, and the inhibition was enhanced with increasing concentration. Compared to the low dose, the high dose but not the medium dose had stronger inhibitory effects with a statistical difference (Figure 2B). Western blot results showed that trifolirhizin blocked the promoting effect of M5 on the expression of PCNA protein in HaCaT keratinocytes in a dose-dependent manner. Pairwise comparisons indicated statistical differences between the three doses (Figure 2C). The changes of cell cycle distribution of HaCaT keratinocytes induced by M5, including shortening of G1 phase and prolongation of S phase, were reversed after treatment with trifolirhizin in a dose-dependent manner. The strongest inhibitory effects on the ratio of S phase were found with the high dose, followed by the medium dose, with statistical differences (Figure 2D). These results indicated that trifolirhizin inhibited the hyperproliferation of psoriasis-like keratinocytes by altering the distribution of the cell cycle.

### Trifolirhizin inhibited excessive synthesis and secretion of IL-8 and IL-12 induced by M5 in human HaCaT keratinocytes

**Figure 3:**
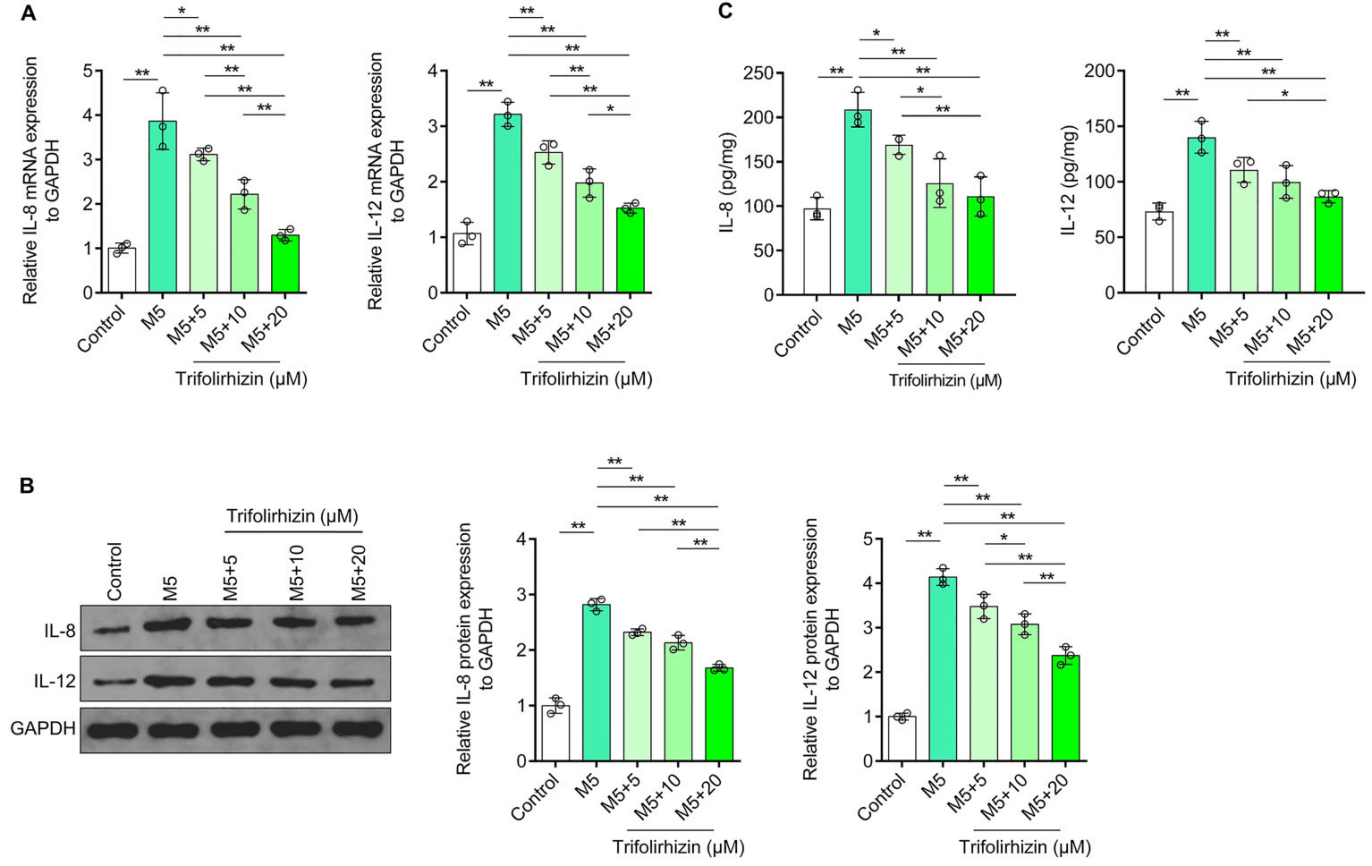
Effects of trifolirhizin on excessive synthesis and secretion of inflammatory cytokines interleukin (IL)-8 and IL-12 in M5-mediated HaCaT keratinocytes treated with trifolirhizin at different concentrations (5-20 μM). **A**, mRNA levels of IL-8 and IL-12 detected by qRT-PCR, using GAPDH as the internal reference. **B**, Western blot image and analysis of IL-8 and IL-12, with GAPDH as the internal reference. **C**, IL-8 and IL-12 determined by ELISA, with protein concentration as the internal reference. Data are reported as means±SD. *P<0.05, **P<0.01; ANOVA.

The results of qRT-PCR showed that compared with the control group, M5 treatment significantly increased the mRNA levels of IL-8 and IL-12 in HaCaT keratinocytes (Figure 3A). The results of western blot were similar to those of qRT-PCR, that is, M5 treatment significantly increased the protein expression of IL-8 and IL-12 in HaCaT keratinocytes (Figure 3B). ELISA assay showed that the secretion of IL-8 and IL-12 increased in human HaCaT keratinocytes exposed to M5 (Figure 3C). The results indicated that M5 promoted the synthesis and secretion of IL-8 and IL-12 in HaCaT keratinocytes. Additionally, intervention with all doses of trifolirhizin inhibited IL-8 and IL-12 mRNA levels in HaCaT keratinocytes induced by M5 treatment. The inhibitory effects of high concentrations of trifolirhizin were strongest on the mRNA level of IL-8 and IL-12 of HaCaT keratinocytes (Figure 3A). All three doses of trifolirhizin also inhibited M5-induced up-regulation of IL-8 and IL-12 protein in HaCaT keratinocytes, which was dose-dependent (Figure 3B). Furthermore, trifolirhizin also inhibited the secretion of IL-8 and IL-12 of HaCaT keratinocytes exposed to M5, and the inhibitory effect was enhanced with increasing trifolirhizin concentration (Figure 3C). These data suggested that trifolirhizin could inhibit the excessive synthesis and secretion of IL-8 and IL-12 in HaCaT keratinocytes exposed to M5 in a dose-dependent manner.

### Trifolirhizin blocked the inhibition of autophagy and AMPK-mTOR signaling pathway in psoriasis-like mouse and cell models

**Figure 4:**
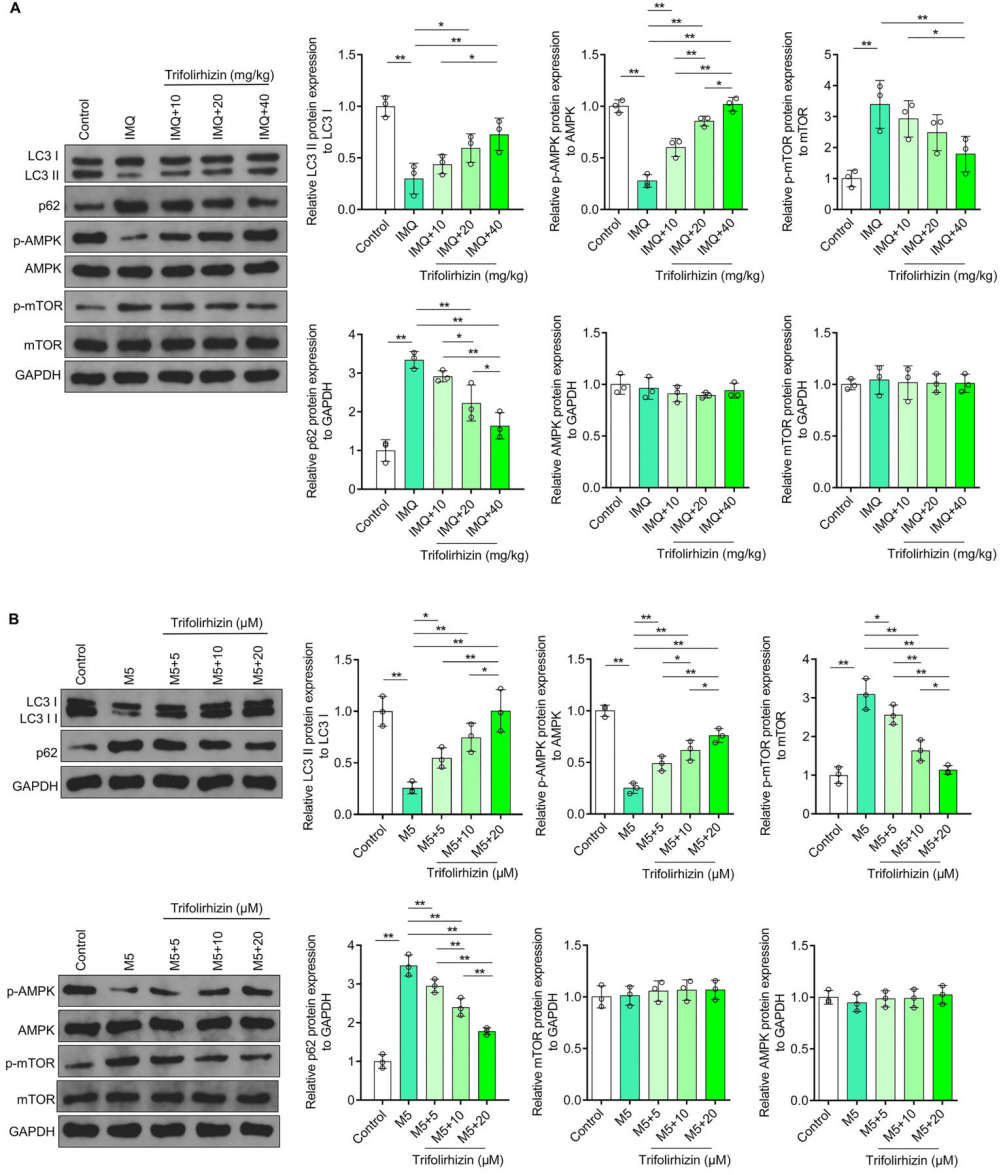
Effects of different doses (5, 10, 20, and 40 mg/kg) of trifolirhizin on autophagy level and AMPK-mTOR signaling pathway in the epidermal layer of psoriasis-like mouse models and M5-induced HaCaT keratinocytes. **A**, Western blot images and analysis of trifolirhizin and vehicle cream or cream containing 5% imiquimod (IMQ) on the LC3 II/I ratio and on the levels of p62, p-AMPK, AMPK, p-mTOR, and mTOR, with GAPDH as the internal reference. **B**, Western blot analysis and images of LC3 II/I ratio and p62, p-AMPK, AMPK, p-mTOR, and mTOR levels in HaCaT keratinocytes, using GAPDH as the internal reference. Data are reported as means±SD. *P<0.05, **P<0.01; ANOVA.

It has been reported that autophagy in psoriasis-like mouse models is inhibited, and activation of autophagy can improve psoriasis-like lesions (28). Therefore, western blot was used to detect the ratio of autophagy marker LC3II and LC3I and the content of autophagy receptor p62 in skin lesions in psoriasis-like mouse models. The results showed that LC3II/I ratio in the epidermal layer of the skin tissue of psoriasis-like mice was significantly decreased and p62 content was increased compared with the normal control mice. Moreover, the regulatory effects of trifolirhizin gradually increased with the increase of concentration. There was a significant difference of the LC3II/I ratio between the high concentration treatment group and the low concentration treatment group. The high and medium doses had stronger effects on the expression of p62 than the low dose, and the effects of the high dose was stronger than that of the medium dose (Figure 4A). It has been reported that the autophagy induction effect of trifolirhizin on colorectal cancer cells is dependent on AMPK-mTOR pathway activation (25). Western blot analysis showed that the phosphorylation level of AMPK was decreased and the phosphorylation level of mTOR was increased in the epidermal layer of the skin lesions from IMQ-induced mice, which could be blocked by trifolirhizin. Among them, the effects of high-concentration trifolirhizin were significantly stronger than that of the low concentration (Figure 4A).

Western blot assay was also used to explore the effects of M5 and trifolirhizin on autophagy and AMPK/mTOR signaling pathway in HaCaT keratinocytes. In HaCaT keratinocytes exposed to M5, the LC3II/I ratio decreased and the expression of p62 increased, which could be reversed by trifolirhizin at all concentrations. The effects of high-concentration trifolirhizin on the LC3II/I ratio and p62 level in M5-treated HaCaT keratinocytes were significantly stronger than that of the low and medium concentrations (Figure 4B). Additionally, the M5-induced reduction of AMPK phosphorylation and the increase of mTOR phosphorylation were reversed by trifolirhizin. Among the three treatment concentrations, high concentration trifolirhizin had the strongest regulatory effect on p-AMPK and p-mTOR content with a statistical difference (Figure 4B). These data suggested that trifolirhizin effectively prevented the inhibition of autophagy and AMPK-mTOR signaling pathway in the epidermal layer from skin lesions of psoriasis-like mice and M5-induced HaCaT keratinocytes in a dose-dependent manner.

### Autophagy inhibitors blocked the effect of trifolirhizin on the hyperproliferation induced by M5 in human HaCaT keratinocytes

**Figure 5:**
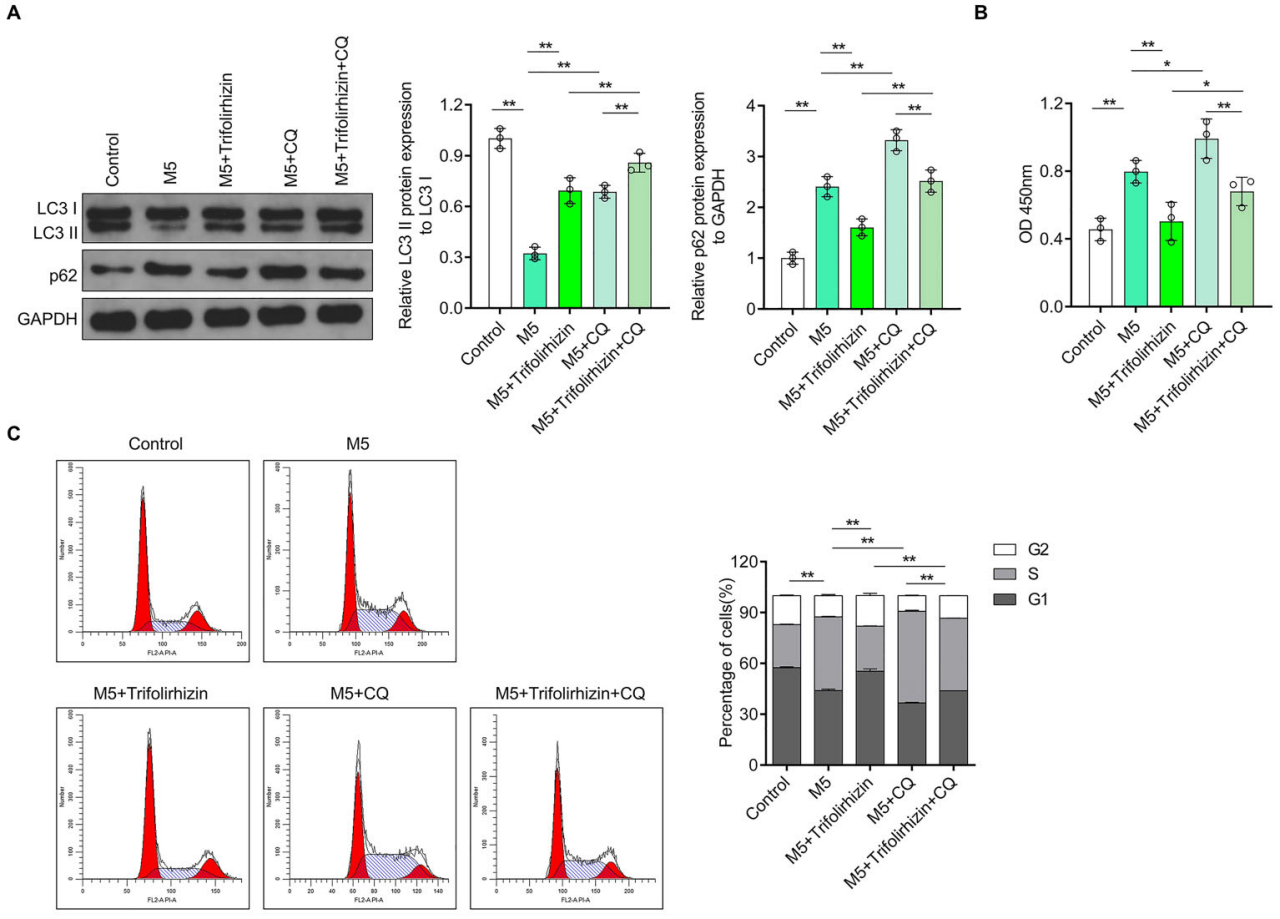
Effects of autophagy inhibitor chloroquine (CQ) (40 μM) and trifolirhizin (20 μM) on M5-mediated HaCaT keratinocytes hyperproliferation. **A**, Western blot image and analysis of LC3II/I ratio and p62 protein expression using GAPDH as the internal reference. **B**, Cell viability by CCK-8. **C**, Propidium iodide staining combined with flow cytometry was used to determine the cell cycle distribution. The groups were compared according to G1 phase. Data are reported as means±SD. *P<0.05, **P<0.01; ANOVA.

Western blot results showed that CQ inhibited the M5-mediated reduction of LC3II/I ratio and further promoted the increase of p62 content induced by M5 in HaCaT keratinocytes. CQ also enhanced the improving effect of trifolirhizin on the reduction of LC3II/I ratio and blocked the inhibitory effect of trifolirhizin on the up-regulation of p62 content in M5-mediated HaCaT keratinocytes (Figure 5A). CQ inhibited autophagic flux by inhibiting autophagosome-lysosome fusion (29). These results indicated that the blocking effect of trifolirhizin on M5-mediated autophagy inhibition of HaCaT keratinocytes could be inhibited by CQ.

The results of CCK-8 showed that CQ could further aggravate the M5-mediated cell viability up-regulation in HaCaT keratinocytes. The inhibitory effect of trifolirhizin on the cell viability of HaCaT keratinocytes induced by M5 could be blocked by CQ (Figure 5B). In addition, the G1 phase shortening and S phase prolongation effects of M5 on HaCaT keratinocytes were further increased after CQ treatment. Trifolirhizin inhibited the change of cell cycle distribution induced by M5 in HaCaT keratinocytes, and this regulatory effect was reversed by CQ (Figure 5C). These results indicated that the improvement of trifolirhizin on the hyperproliferation of HaCaT keratinocytes induced by M5 was dependent on autophagy.

### Autophagy inhibitors blocked the improving effect of trifolirhizin on excessive synthesis and secretion of IL-8 and IL-12 induced by M5 in human HaCaT keratinocytes

**Figure 6:**
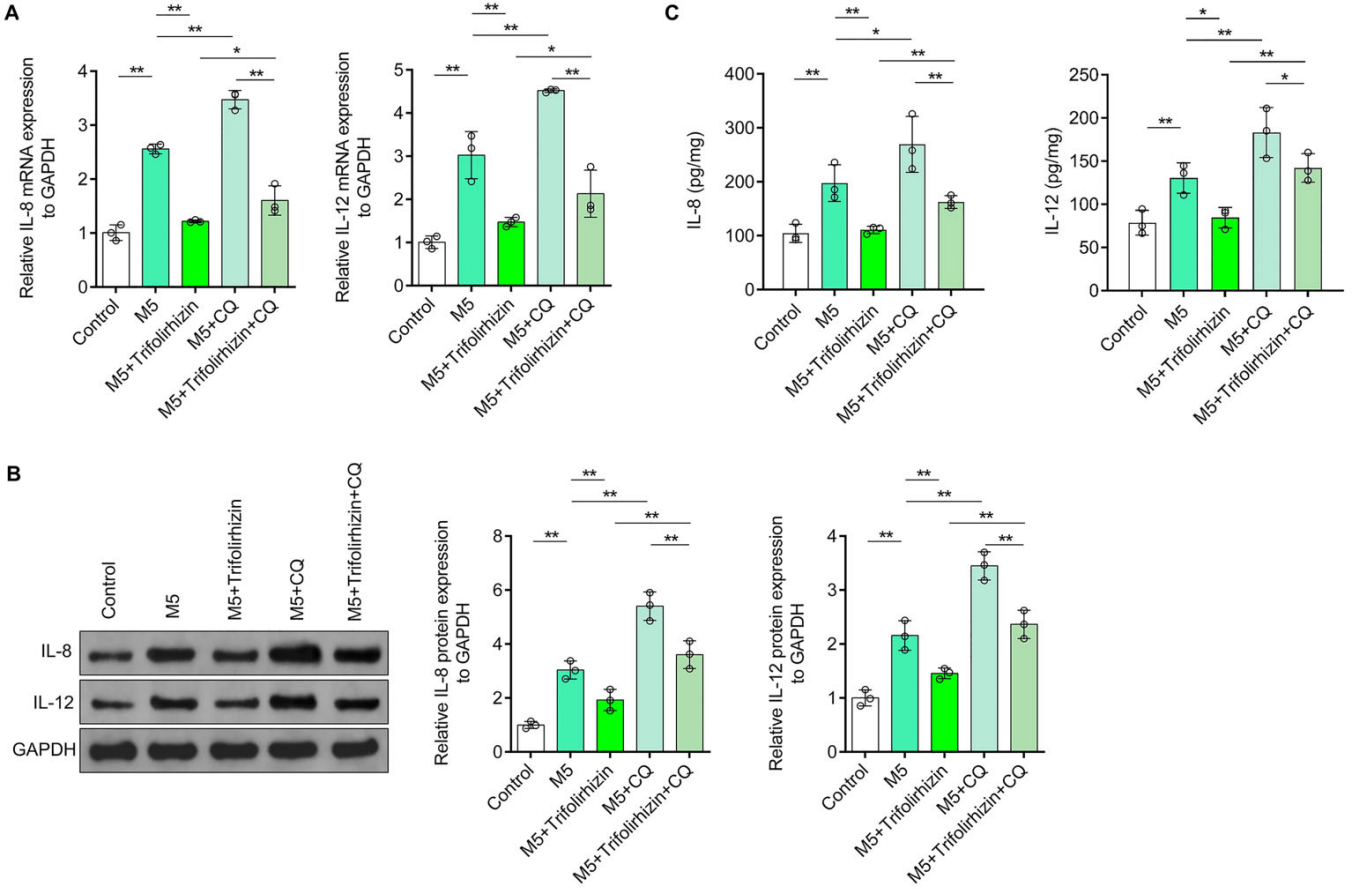
Effects of autophagy inhibitor chloroquine (CQ) (40 μM) and trifolirhizin (20 μM) on excessive synthesis and secretion of interleukin (IL)-8 and IL-12 in M5-induced HaCaT keratinocytes. **A**, mRNA levels of IL-8 and IL-12 detected by qRT-PCR, using GAPDH as the internal reference. **B**, Western blot images and analysis of protein levels of IL-8 and IL-12, with GAPDH as the internal reference. **C**, IL-8 and IL-12 in the supernatant determined by ELISA, using the protein concentration as the internal reference. Data are reported as means±SD. *P<0.05, **P<0.01; ANOVA.

The results of qRT-PCR showed that CQ could enhance M5-mediated increase of IL-8 and IL-12 mRNA levels in HaCaT keratinocytes. The inhibitory effect of trifolirhizin on the mRNA levels of IL-8 and IL-12 in HaCaT keratinocytes exposed to M5 were reversed by CQ (Figure 6A). The results of western blot were similar to those of qRT-PCR. Compared with the control group, IL-8 and IL-12 protein levels in HaCaT keratinocytes in the M5 treatment group were significantly increased and increased further after CQ treatment. Trifolirhizin blocked M5-induced up-regulation of IL-8 and IL-12, which could be inhibited by CQ (Figure 6B). ELISA results showed that CQ treatment aggravated the secretion of IL-8 and IL-12 induced by M5 in HaCaT keratinocytes. Trifolirhizin-mediated IL-8 and IL-12 inhibition in M5-treated HaCaT keratinocytes was reversed by CQ (Figure 6C). These results indicated that trifolirhizin inhibited excessive synthesis and secretion of inflammatory cytokines IL-8 and IL-12 in M5-induced HaCaT keratinocytes through autophagy.

## Discussion

Keratinocyte hyperproliferation is important to the pathogenesis of psoriatic skin lesions. Trifolirhizin has an anti-proliferation effect and can inhibit the growth of gastric cancer cells (17), colorectal cancer cells (25), lung cancer cells, and ovarian cancer cells (15). In this study, we found that trifolirhizin improved psoriasis-like skin lesions, including erythema, scales, and epidermal thickening, with high concentrations (40 mg/kg) having the best effect. For *in vitro* models of psoriasis-like keratinocytes induced by M5, trifolirhizin (5-20 μM) was found to inhibit the hyperproliferation of HaCaT keratinocytes in a dose-dependent manner. In this concentration range, trifolirhizin was not toxic to HaCaT keratinocytes in normal medium without M5 stimulation. Therefore, trifolirhizin has the potential to be used as anti-psoriasis treatment.

Further analysis revealed that hyperproliferation in psoriasis-like keratinocytes was linked to a shortened G1 phase and prolonged S phase of the cell cycle. Trifolirhizin treatment reversed these changes by extending the G1 phase and shortening the S phase. In conclusion, trifolirhizin mitigated hyperproliferation and psoriasis-like skin lesions by modulating cell cycle distribution.

It has been reported that trifolirhizin was involved in the inhibition of inflammation. For example, trifolirhizin regulates the balance of T helper 17 cells and regulatory T cells in dextran sodium sulfate-induced colitis mice (30). Trifolirhizin dose-dependently inhibited lipopolysaccharide-induced expression of pro-inflammatory cytokines in macrophages, including TNF-α and IL-6 (15). In this study, we found excessive synthesis and secretion of IL-8 or IL-12 in the epidermal tissue of skin lesions from IMQ-induced psoriasis-like mouse models and M5-induced psoriasis-like keratinocytes, which could be inhibited by trifolirhizin. IL-8, mainly produced by keratinocytes, is involved in the proliferation of keratinocytes, neutrophil infiltration, and angiogenesis in psoriasis (8,31). IL-12 is one of the two subunits of the pro-inflammatory factors IL-23 and IL-23B (p40), which promotes Th17 cell differentiation (32). These results suggested that trifolirhizin can inhibit the excessive synthesis and secretion of IL-8 and IL-12 in keratinocytes, thereby improving inflammation and alleviating psoriasis. Whether trifolirhizin inhibits Th17 cell differentiation by inhibiting the secretion of IL-8 and IL-12 in keratinocytes remains to be further studied.

Psoriatic lesions are closely related to autophagy inhibition. Autophagy levels are decreased in the skin tissue of psoriasis-like animal models, while autophagy inducers, such as rapamycin, can activate autophagy and improve skin thickening in psoriasis-like mouse models (28). PSORI-CM02 improves psoriasis by reversing the inhibition of autophagy in skin tissues and keratinocytes (33). Enhanced IL-8 synthesis in keratinocytes is associated with impaired autophagy (34,35). Trifolirhizin can induce autophagy and promote colorectal cancer cell death (25). In this study, we found that the autophagy level was inhibited in the epidermal tissue of psoriasis-like skin lesions and *in vitro* keratinocyte models, which could be reversed by trifolirhizin. The autophagy inhibitor CQ could further increase the proportion of the S1 phase, the cell viability, and the synthesis and secretion of IL-8 and IL-12 of keratinocytes treated with M5. In addition, CQ could also block the improvement of trifolirhizin on keratinocyte hyperproliferation and excessive synthesis and secretion of IL-8 and IL-12 in M5-mediated HaCaT keratinocytes. To summarize, trifolirhizin can reverse autophagy inhibition, keratinocyte hyperproliferation, and excessive synthesis and secretion of IL-8 and IL-12 leading to a reversal of psoriasis-like skin lesions. In addition, impaired differentiation is associated with impaired autophagy (36). The imbalance in keratinocyte proliferation and differentiation homeostasis is closely related to the occurrence of psoriasis-like lesions (37). Whether trifolirhizin can improve the abnormal differentiation of psoriasis-like keratinocytes and whether it is dependent on autophagy needs further investigation.

The AMPK-mTOR pathway is one of the upstream signaling pathways that regulate autophagy activation. AMPK can enhance the phosphorylation levels of Ser317 and Ser777 in ULK1, directly activate ULK1, and induce autophagy (38). AMPK can also inhibit the phosphorylation of mTOR and block the promoting effect of mTOR on the phosphorylation of Ser757 in ULK1, prevent the inactivation of ULK1 and the interaction disorder between ULK1 and AMPK, and ultimately activate autophagy (38). It has been reported that AMPK activation is impaired in epidermal tissue of psoriatic skin lesions in humans, and inhibition of AMPK can lead to abnormal keratosis in mice (39). The inhibition of metformin-mediated keratinocyte proliferation is associated with increased phosphorylation of AMPK (40). In addition, the autophagy induction effect of trifolirhizin on colorectal cancer cells is related to the activation of AMPK-mTOR pathway (25). In this study, we found that trifolirhizin inhibited the decrease of AMPK phosphorylation and the increase of mTOR phosphorylation in epidermal tissue in psoriasis-like skin lesions and keratinocytes. These results suggest that the reversal effect of trifolirhizin on psoriasis-like skin lesions and keratinocytes hyperproliferation may depend on the inhibition of AMPK-mTOR signaling pathway-mediated impaired autophagy. However, whether the regulatory effect of trifolirhizin on keratinocyte hyperproliferation and excessive synthesis and secretion of IL-8 and IL-12 depends on the AMPK-mTOR signaling pathway remains to be confirmed.

There are some limitations in this study. Firstly, the expression of IL-8 and IL-12, activation level of autophagy, and AMPK-mTOR signaling pathway were not analyzed in the epidermal skin lesions from patients with psoriasis. Secondly, the safety of trifolirhizin in mice, including liver, kidney, and body weight and other organs, were not investigated. Moreover, intraperitoneal injection of trifolirhizin was found to improve psoriasis-like mouse models in this investigation, but the effects of smearing trifolirhizin on psoriasis-like lesioned skin is not clear. If smearing trifolirhizin can improve psoriasis-like lesioned skin in mice without toxicity or side effects, smearing trifolirhizin may have potential in the treatment of patients with psoriasis. Lastly, the inhibitors targeting autophagy and AMPK-mTOR pathway combined with trifolirhizin were not applied to psoriasis-like mouse models to confirm the role of autophagy and AMPK-mTOR signaling pathway in the improvement by trifolirhizin on the psoriasis-like process. However, we will investigate the effects of trifolirhizin on psoriatic skin lesions of patients and continue to study the regulatory mechanism through which trifolirhizin regulates AMPK-mTOR pathway in psoriasis-like mouse models and keratinocytes.

## Conclusions

Trifolirhizin can improve psoriasis-like keratinocyte hyperproliferation and excessive synthesis and secretion of inflammatory cytokines IL-8 and IL-12, thereby alleviating psoriasis lesions, probably dependent on reversal of impaired autophagy induced by AMPK-mTOR signaling pathway inhibition. Trifolirhizin may be a potential therapeutic agent for psoriasis but needs further investigation.
